# Density Functional Theory for Steady-State Nonequilibrium Molecular Junctions

**DOI:** 10.1038/srep15386

**Published:** 2015-10-16

**Authors:** Shuanglong Liu, Argo Nurbawono, Chun Zhang

**Affiliations:** 1 Department of Physics and Graphene Research Centre, National University of Singapore, 2 Science Drive 3, Singapore, 117542; 2 Department of Chemistry, National University of Singapore, 3 Science Drive 3, Singapore, 117543.

## Abstract

We present a density functional theory (DFT) for steady-state nonequilibrium quantum systems such as molecular junctions under a finite bias. Based on the steady-state nonequilibrium statistics that maps nonequilibrium to an effective equilibrium, we show that ground-state DFT (GS-DFT) is not applicable in this case and two densities, the total electron density and the density of current-carrying electrons, are needed to uniquely determine the properties of the corresponding nonequilibrium system. A self-consistent mean-field approach based on two densities is then derived. The theory is implemented into SIESTA computational package and applied to study nonequilibrium electronic/transport properties of a realistic carbon-nanotube (CNT)/Benzene junction. Results obtained from our steady-state DFT (SS-DFT) are compared with those of conventional GS-DFT based transport calculations. We show that SS-DFT yields energetically more stable nonequilibrium steady state, predicts significantly lower electric current, and is able to produce correct electronic structures in local equilibrium under a limiting case.

As the trend of miniaturization continues, the next generation of electronic devices will likely be downsized to molecular scale. Since the first molecular device, the so-called Avram-Ratner (AR) rectifier^1^, numerous molecular electronic devices with unusual properties such as high-performance transistors^2^,^3^,^4^, light driven switches^5^,^6^,^7^, and novel spintronics devices^8^,^9^,^10^ have been suggested experimentally and/or theoretically. Previous studies have clearly shown that many exciting features of these devices are closely related to the intrinsic electronic properties of the molecular junction. Understanding the electronic properties of the molecular junction under the operating conditions now has become the central issue in the theoretical modeling of these devices.

Due to highly reduced dimensions, the properties of molecular devices are often strongly size-dependent and very sensitive to their chemical environment. A reliable and efficient parameter-free first-principles modeling technique is therefore highly desired. There are two major difficulties in atomistic first-principles modeling of these devices. Firstly, the device is connected with two electrodes so that the whole system is not periodic and the so-called scattering boundary conditions (SBCs) have to be considered. Secondly, since the molecular devices always operate under a finite bias, the bias-induced nonequilibrium effects need to be correctly taken into account, which presents a great challenge to the modeling. The first difficulty has been resolved. It has been shown that the mean-field equation from density functional theory (DFT) with SBCs can be solved either by plane-wave based methods^11^ or by non-equilibrium Green’s functions’ (NEGF) techniques^12^. The so-called DFT + NEGF method also included part of nonequilibrium effects and was then implemented with various computational schemes^13^,^14^,^15^ and widely applied in studying all kinds of nonequilibrium molecular scale devices. Over the last decade, the DFT + NEGF method has achieved great success in terms of providing insights into experiments and inspiring novel devices. While, the aforementioned second difficulty still remains because DFT is a theory for ground-state (GS) properties. The applicability of DFT for nonequilibrium systems is questionable. In fact, several recent works^16^,^17^ have argued that when a system is under a finite bias, the basis of GS-DFT, the first Hohenberg-Kohn theorem (HKT)^18^ may not be applicable any more.

In our previous work, without rigorous proof, we suggested that additional degrees of freedom (could be an additional density) besides the total electron density are needed to determine the properties of a nonequilibrium quantum system^16^. In this paper, starting from the Hershfield’s nonequilibrium quantum statistics^19^, we derive a density functional theory for the steady-state (SS) properties of molecular junctions under a finite bias. We prove that with given two densities, the total electron density and the density of current-carrying electrons, the Hamiltonian of an effective equilibrium system (that can be mapped to the desired steady-state nonequilibrum junction) is uniquely determined, and in turn the steady-state properties of the corresponding nonequilibrium system are also determined, confirming that GS-DFT is in principle incorrect for nonequilibrium systems. A self-consistent mean-field approach based on the two densities is then derived by minimizing the energy of the effective equilibrium system. The theory is called SS-DFT (comparing to GS-DFT) in this paper. We apply the theory to a carbon-nanotube (CNT)/Benzene junction. Compared with the standard GS-DFT based DFT + NEGF method, the SS-DFT gives significantly lower energies at all biases, and importantly, we show that for a limiting case (which will be shown later) for which the electric current is negligible, the SS-DFT predicts the correct electronic properties of the molecular center while the DFT + NEGF does not.

## Model and Theory

**Figure 1:**
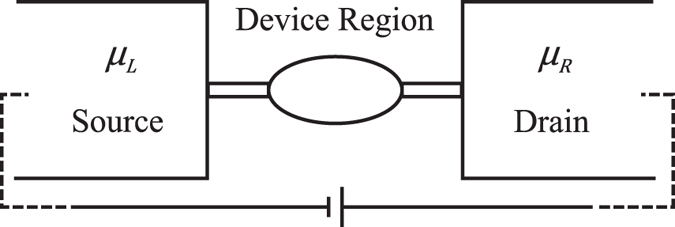
Model of a molecular junction under a finite bias. The model consists of source, drain and a molecular scale device region. The source and drain are assumed to be non-interacting and in local equilibrium.

We illustrate in Fig. 1 the model for a molecular junction under a finite bias. The model consists of two reservoirs that are in their own equilibrium (the source and drain) and a molecular scale device region. We assume mean-field Hamiltonians in two reservoirs so that two chemical potentials, *μ*_*L*_ and *μ*_*R*_, can be defined for the source and drain, respectively. Electrons in the device region, however, are interacting. Without losing generality, we assume *μ*_*L*_ ≥ *μ*_*R*_. The bias voltage is defined as *eV*_*b*_ = *μ*_*L*_ − *μ*_*R*_. The overall Hamiltonian for such an open system can be written as 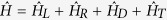 consisting of the Hamiltonian for the non-interacting left (right) reservoir 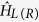, the interacting device region 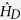, and the tunneling term 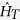.

When the nonequilibrium system is in steady state, Hershfield^19^ showed that the nonequilibrium ensemble average of any physical observable can be calculated using an equilibrium-like statistics,


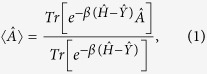


where 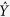 accounts for nonequilibrium distribution, and can be written as following^20^,


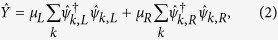


in which the creation operator 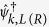 creates an electron incident from the left (right) reservoir. Defining two number operators 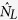 and 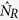, 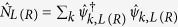, the distribution operator becomes 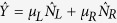. Hershfield’s theory provides a very attractive way for investigating steady-state properties of nonequilibrium systems without the need of complicated time-dependent perturbation. However, due to the difficulty of working out the distribution operator 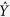 from a many-body Hamiltonian, the application of the theory has been rather limited. The purpose of this paper is to derive a DFT-like first-principle method based on Hershfield’s nonequilibrium statistics that can be applied to realistic systems.

We introduce another set of number operators, 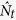 and 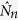, defined as 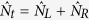 and 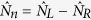. Eq. 1 can then be rewritten as,


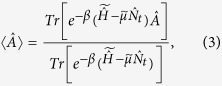


where 
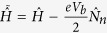
 and 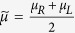. Eq. 3 clearly shows that the steady-state properties of the nonequilibrium system can be obtained from a corresponding effective equilibrium system with the Hamiltonian 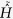 and the chemical potential 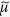.

Apparently, 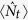 and 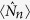 have physical meanings of the total number of electrons and the number of current-carrying electrons, respectively. After transforming the number operators to real space by Fourier transform, two electron densities, ρ_*t*_(*r*) and ρ_*n*_(*r*), can be defined (Eq. 4). The integration of these densities in real space yield 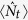 and 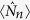, respectively.


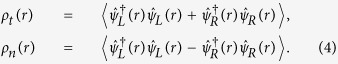


We are now ready to derive the first HKT-like theorem for the nonequilibrium steady state of the system. We will prove that the two densities, ρ_*t*_(*r*) and ρ_*n*_(*r*), uniquely determine the Hamiltonian of the effective equilibrium system 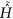 and in turn the steady-state properties of the nonequilibrium system (Eq. 3). The proof is given in the following discussions. In general, the Hamiltonian 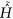 reads as follows,


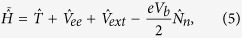


where the kinetic 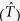 and e-e interaction 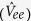 terms are universal for all systems. Unlike the ground-state case where the Hamiltonian is completely determined by the external potential 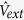, the effective-equilibrium Hamiltonian here 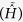 is determined by three terms, the external potential 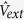, the bias voltage *V*_*b*_, and the nonequilibrium number operator 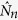. The bias voltage *V*_*b*_ is a global term that is associated with boundary conditions provided by two reservoirs and cannot be determined by total electron density alone as discussed in our previous work^16^ so that the first HKT for GS-DFT is not applicable here. In order to prove that two densities determine everything including *V*_*b*_, we first assume that there are two different effective equilibrium Hamiltonians, 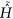 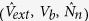 and 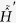 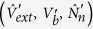, which have two ground states 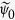 and 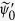 respectively and bear the same set of densities ρ_*t*_ and ρ_*n*_. Then we have,


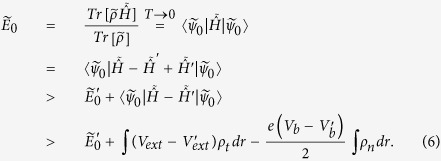


In above derivations, the temperature is set to zero (*T* → 0), so the equilibrium ensemble average becomes a ground-state integral. 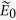 and 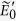 are ground-sate energies of 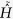 and 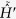, respectively. 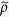 is the distribution function defined in Eq. 3. We can also start from the ensemble average of 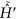 and similarly,


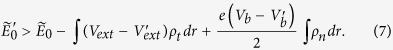


Combining Eq. 6 and Eq. 7, we get


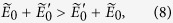


which cannot be true. Thus ρ_*t*_ and ρ_*n*_ determine 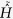 up to a trivial additive constant, and in turn, determine the ensemble average of any physical observables (Eq. 3) of the nonequilibrium steady state. Note that the ground state of the effective equilibrium is assumed to be non-degenerate in the above derivations.

The next step is to derive a mean-field approach to calculate the two electron densities. For this purpose, we write the ground-state energy of the effective equilibrium 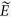 as a functional, 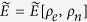, where ρ_*e*_ ≡ ρ_*t*_ − ρ_*n*_. By generalizing the local density approximation (LDA) to nonequilibrium cases, the energy functional becomes,


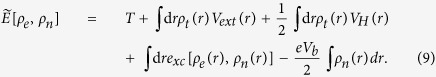


In Eq. 9, *T* is the kinetic energy, *V*_*H*_ is the Hartree potential that should be calculated under proper open-system boundary conditions, and *e*_*xc*_ is the exchange correlation energy density of nonequilibrium uniform electron gas.

To make the calculations feasible, we assume that the electron densities in two mean-field equilibrium reservoirs are not affected by interactions in the device region due to the screening in contacts, which is consistent with the conventional transport modeling^13^,^15^. As a consequence, the electron densities in two reservoirs can be treated as boundary conditions and the variations of the densities can be done only in the device region. Before applying the variational principle to Eq. 9, following the spirit of the Kohn-Sham approach of GS-DFT, we introduce a non-interacting reference system of which the single-electron states bear real equilibrium and current-carrying electron densities in the device region,


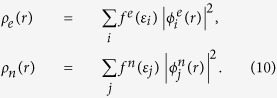


In the equations above, single-electron state 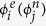 is normalized in the device region; *f*^*e*^ and *f*^*n*^ together specify the nonequilibrium distribution


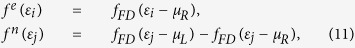


where *f*_*FD*_ is the Fermi-Dirac distribution function at zero temperature. When an infinitesimal variation is applied to any state *ϕ* (either 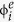 or 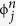) in the reference system,


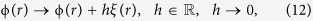


we have the variational condition 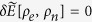.

In order to make the variational principle sensible, we add a constraint that the total number of electrons in the device region is conserved. There are also two boundary conditions arising from the assumption that the variations are only done in the device region, which read (∂Ω_*D*_ denotes the boundaries of the device region)


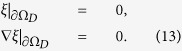


To solve the constrained variational problem, we use the method of Lagrange multipliers. Define


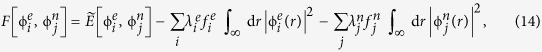


where 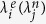 are real and 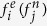 are the occupation numbers for equilibrium (current-carrying) electrons. Let


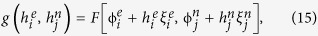


the variational condition then becomes


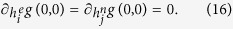


After some straightforward derivations, we arrive at the following two mean-field equations, one for current-carrying electrons and another one for equilibrium electrons that do not contribute to current,


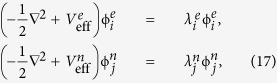


where the two effective potentials are


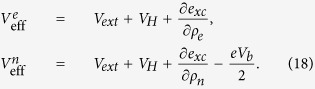


As shown in Eq. 18, two effective potentials differ in the third term, the so-called exchange-correlation potential *V*_*xc*_. The effective potential for current-carrying electrons has an additional term which is proportional to bias voltage. Note that Eq. 18 can be easily generalized to include generalized gradient approximation (GGA) (See supporting information). The two mean-field equations (Eq. 17) are coupled with each other through the exchange-correlation functional that depends on both ρ_*e*_ and ρ_*n*_. These equations have to be solved self-consistently (see the self-consistent procedure in Figure S1 of supporting information). By assuming that the correlation functional is the same as GS case and using the nonequilibrium exchange functional we previously derived^16^,^17^, the SS-DFT can be applied to real molecular junctions.

## Results and Discussions

**Figure 2:**
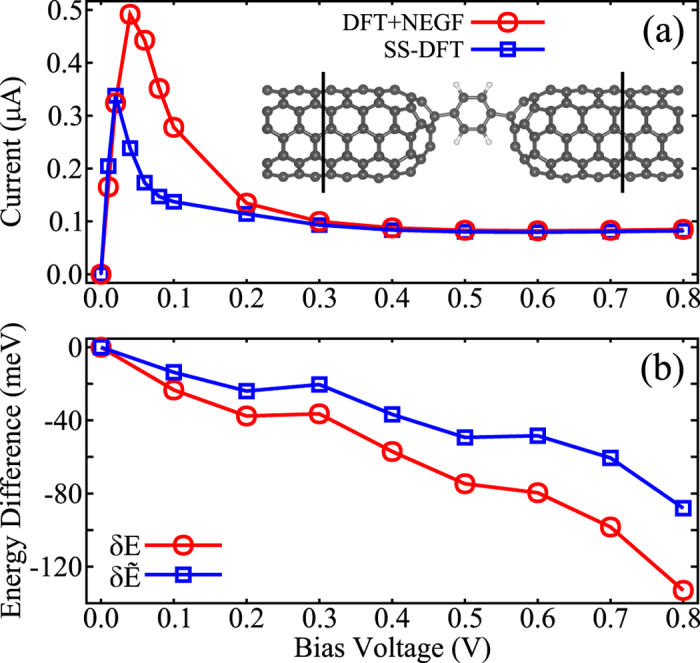
(**a**) Current-Voltage characteristics of CNT-Benzene-CNT junction from SS-DFT and DFT + NEGF calculations. Inset: Atomic structure of the junction. (**b**) Energy difference between SS-DFT and DFT + NEGF methods defined as δ*E* = *E*_SS−DFT_ − *E*_DFT+NEGF_. Both δ*E* and 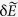 are presented. The SS-DFT always gives lower energies when the bias voltage is not zero.

We have implemented the SS-DFT into the widely used computational package SIESTA^21^. The theory is then applied to a CNT-benzene-CNT junction that can be fabricated in lab with radical chemistry. Energetics and I-V characteristics from SS-DFT are compared to those obtained from the conventional DFT + NEGF approach via the function TranSIESTA^15^. As shown in the inset of Fig. 2a, the CNT-benzene-CNT molecular junction consists of two semi-infinite metallic (5, 5) CNTs and a benzene molecule in between. In order to form good contacts, two H atoms in the benzene molecule are taken away (which can be done in chemical synthesis). Both CNTs are closed at one end with a *C*_30_ cap. The atomic structures of CNTs, the device region and the distance between two leads are optimized with full quantum calculations. In all calculations, we used single-*ζ* basis set and PBE exchange-correlation functional^22^ corrected by the nonequilibrium exchange energy^16^,^17^. In the Brillouin zone, 1 × 1 × 30 k-point sampling for lead calculations is employed. The energy and force (for structure relaxation) convergence criteria are set to 10^−4^ *eV* and 0.04 *eV*/Å, respectively.

Two methods, SS-DFT and DFT + NEGF, yield similar I-V curves for the junction under study except that between bias voltages from 0.05 and 0.2 V, SS-DFT predicts significantly lower currents. In Fig. 2b, we plot the energy difference between two methods. Note that *E* and 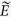 are ensemble averages of 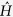 and 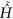, respectively. Clearly, SS-DFT gives lower energies in all bias voltages, which is expected since compared with DFT + NEGF, SS-DFT searches the lowest-energy steady state in a much bigger Hilbert space. For equilibrium situation when ρ_*n*_ = 0, SS-DFT reduces to GS-DFT. Consequently, there is no essential difference between SS-DFT and DFT + NEGF at zero bias. Both methods predict a prominent negative-differential-resistance (NDR) peak around 0.05 V where the SS-DFT peak occurs at a slightly smaller bias than the DFT + NEGF one. This can be understood by the fact that the two methods generate different electronic properties of the device region and in this case, the lowest unoccupied orbital (LUMO) of the benzene molecule calculated from SS-DFT exits the bias window earlier than the one computed from DFT + NEGF (see details in Figure S2 of supporting information), leading to different positions of NDR peaks.

**Figure 3:**
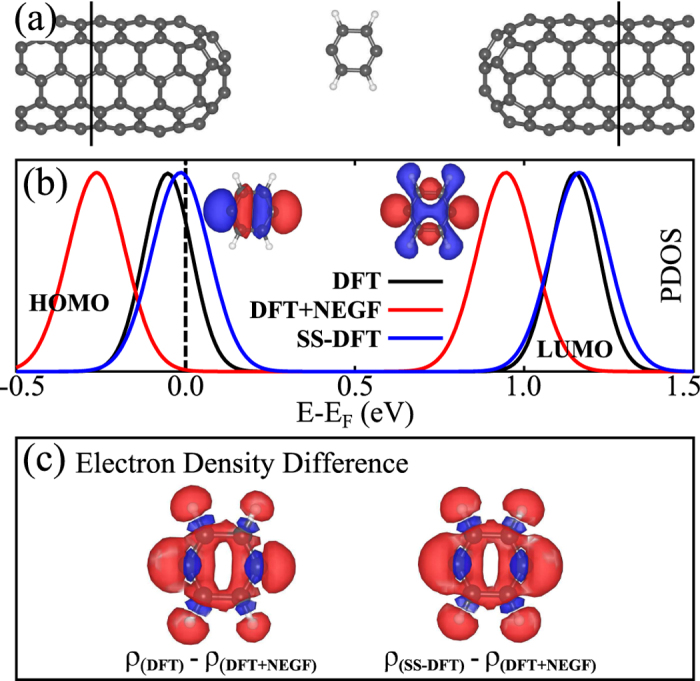
(**a**) The atomic structure of a limiting case where the distance between the center molecule and each of two leads is more than 6 Å. In this case, the center molecule is essentially isolated. (**b**) Projected density of states (PDOS) of the center molecule calculated from three different methods, DFT for zero bias case, DFT + NEGF and SS-DFT for the junction under a bias voltage of 0.5 V. *E*_*F*_ in the figure represents 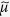. (**c**) The difference in the total electron densities calculated from different methods, δρ = ρ_DFT_ − ρ_DFT+NEGF_ and δρ = ρ_SS−DFT_ − ρ_DFT+NEGF_. The isosurface value is 5 × 10^−5^ Bohr^−3^ in both plots where the red (blue) color denotes positive (negative) value.

We now consider a limiting case of which the electronic properties of a certain part are known and check whether or not the SS-DFT is able to reproduce those known properties. The limiting case is shown in Fig. 3a where the distance between the center molecule and the leads is set to more than 6 Å. In this case, the electric current is essentially zero and the center molecule is decoupled from the two leads. Although the whole system is driven out of equilibrium by the external bias (0.5 V of voltage for this case), the center molecule is in local equilibrium, which means that its properties can be determined by GS-DFT. We therefore perform calculations for the electronic properties of the center molecule using three different methods, the GS-DFT for the junction under zero bias, the SS-DFT and DFT + NEGF for the junction under 0.5 V of bias voltage. In Fig. 3, we show the calculated projected density of states (PDOS) of the center molecule around the Fermi Energy. The SS-DFT agrees with GS-DFT (DFT in the figure) very well, while the DFT + NEGF generates different electronic structures. To further illustrate the difference between different methods, in Fig. 3c, we plot the difference between total electron densities around the center molecule calculated from different methods, δρ = ρ_DFT_ − ρ_DFT+NEGF_ in left and δρ = ρ_SS−DFT_ − ρ_DFT+NEGF_ in right. Clearly, DFT + NEGF yields quite different total electron densities from other two methods, and the densities from DFT and SS-DFT are similar. Note that in GS-DFT calculations, there is no electric field across the molecule, which leads to unsymmetrical δρ in the left panel of Fig. 3c. We also performed a simple GS-DFT calculations with an external electric field (without the presence of two leads), and then the δρ around the molecule between GS-DFT and DFT + NEGF becomes symmetrical (see Figure S3 in supporting information).

## Conclusion

In summary, we present in this paper a density functional theory for the steady state of nonequilibrium molecular junctions. We prove that two electron densities, ρ_*t*_ and ρ_*n*_, uniquely determine the steady-state properties of nonequilibrium quantum systems. Two mean-field equations that are coupled with each other, one for equilibrium electrons and another one for current-carrying electrons, are derived. The theory is applied to a CNT-benzene-CNT junction. Calculations based on the SS-DFT are compared with those from the conventional DFT + NEGF calculations via TranSIESTA. It is found that the SS-DFT always leads to energetically more stable steady states and lower electric currents than conventional GS-DFT based transport calculations. We also show that for a limiting case in which the center molecule is essentially isolated, the SS-DFT is able to predict correct local equilibrium properties. The theory lays a solid ground for atomistic first-principles modeling of steady-state nonequilibrium quantum systems and paves the way for computational understanding and design of complex molecular scale devices.

## Additional Information

**How to cite this article**: Liu, S. *et al.* Density Functional Theory for Steady-State Nonequilibrium Molecular Junctions. *Sci. Rep.* **5**, 15386; doi: 10.1038/srep15386 (2015).

## Supplementary Material

Supporting Information
