# Supplementary material for: Density Functional Theory for Steady-State Nonequilibrium Molecular Junctions
Source: Sci Rep. 2015 Oct 16;5:15386. doi: 10.1038/srep15386 (PMC4608000; doi:10.1038/srep15386)
Supplement: Supporting Information [file srep15386-s1.pdf]

Density Functional Theory for Steady-State Nonequilibrium Molecular Junctions

Shuanglong Liu[1], Argo Nurbawono[1] and Chun Zhang[1,2,*]

[1] *Department of Physics and Graphene Research Centre, National University of Singapore, 2 Science Drive 3, Singapore 117542*

[2] *Department of Chemistry, National University of Singapore, 3 Science Drive 3, Singapore 117543*

When considering GGA, the exchange-correlation energy functional becomes $E[\rho_e, \rho_n, \nabla\rho_e, \nabla\rho_n]$. In this paper, for simplicity, we use the GS-DFT GGA functional in the form $E[\rho_e, \rho_n, \nabla\rho_t]$. Then the exchange-correlation potential become as follows, which are the straightforward generalization of GS-DFT.

$$V_{xc}^{e} = \frac{\partial e_{xc}}{\partial \rho_e} - \boldsymbol{\nabla} \cdot \frac{\partial e_{xc}}{\partial \boldsymbol{\nabla} \rho_t},$$

$$V_{xc}^{n} = \frac{\partial e_{xc}}{\partial \rho_n} - \boldsymbol{\nabla} \cdot \frac{\partial e_{xc}}{\partial \boldsymbol{\nabla} \rho_t}.$$

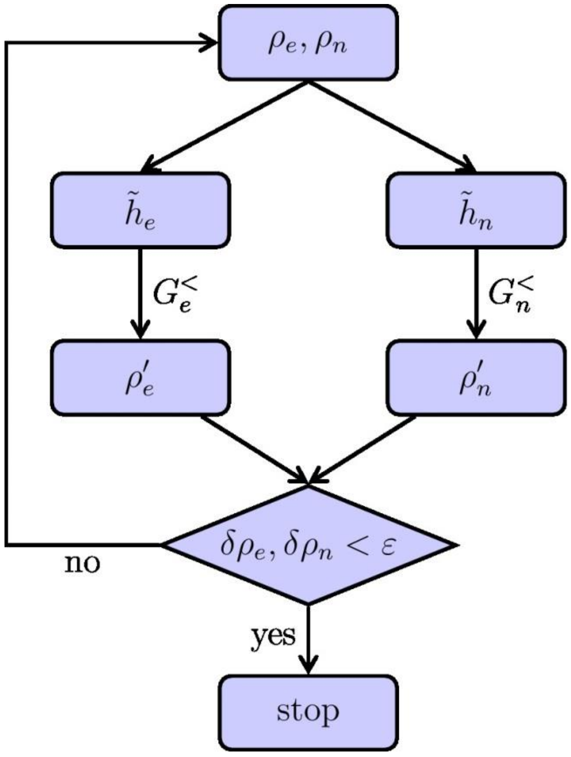

Figure S1 The Self-consistent procedure for solving the SS-DFT mean-field equations. At first, given equilibrium electron density $\boldsymbol{\rho_e}$ and current-carrying (nonequilibrium) electron density $\boldsymbol{\rho_n}$, mean field Hamiltonians, $\tilde{\boldsymbol{h}}_{\boldsymbol{e}}$ for equilibrium electrons and $\tilde{\boldsymbol{h}}_{\boldsymbol{n}}$ for nonequilibrium electrons, are constructed. Then new electron densities $\boldsymbol{\rho}'_{\boldsymbol{e}}$ and $\boldsymbol{\rho}'_{\boldsymbol{n}}$ are computed via lesser Green's functions $\boldsymbol{G}_{\boldsymbol{e}}^{<}$ and $\boldsymbol{G}_{\boldsymbol{n}}^{<}$ which correspond to $\tilde{\boldsymbol{h}}_{\boldsymbol{e}}$ and $\tilde{\boldsymbol{h}}_{\boldsymbol{n}}$ respectively. The calculations are repeated until both densities converge.

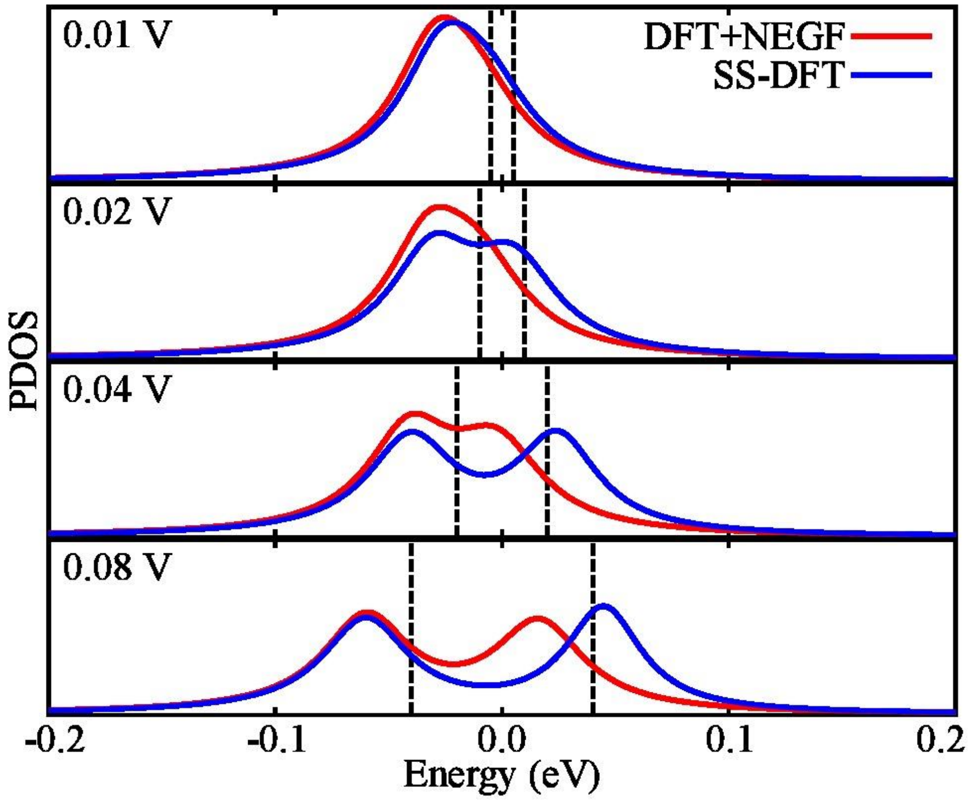

Figure S2 Projected density of states (PDOS) for the center molecule in the CNT-benzene-CNT junction calculated from both SS-DFT and DFT+NEGF at various bias voltages. Two states are degenerate when the bias voltage is small; they split at large bias voltages. As the bias voltage increases, the high energy state (called LUMO in this document) is shifted towards right and the low energy state (HOMO) is shifted towards left. The energy difference between them is close to the difference between left and right chemical potentials. For the SS-DFT calculation, the state with higher energy enters the bias window at 0.02 V and exits the bias window at 0.04 V.

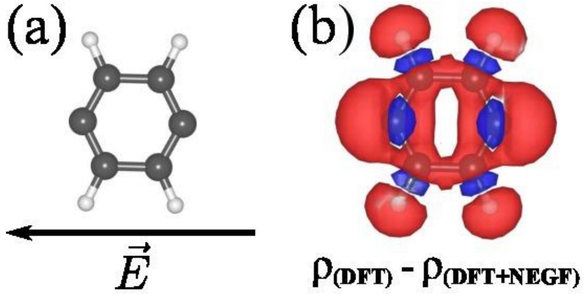

Figure S3 Total electron density difference $\boldsymbol{\delta\rho = \rho_{(DFT)} - \rho_{(DFT+NEGF)}}$. DFT+NEGF calculation is for the CNT-benzene-CNT junction under a bias voltage of $\mathbf{0.5\ V}$. DFT calculation is for an isolated molecule (figure a) under an electric field of strength $\mathbf{0.014\ V/}$Å. This electric field strength equals $\mathbf{0.5\ V}$ divided by the distance between left and right leads of the CNT-benzene-CNT junction. Compared with Fig. 3c in the main text, the electron density difference shown here (figure b) become symmetric indicating that the polarization effects in the two calculations, DFT and DFT+NEGF, cancel each other.
